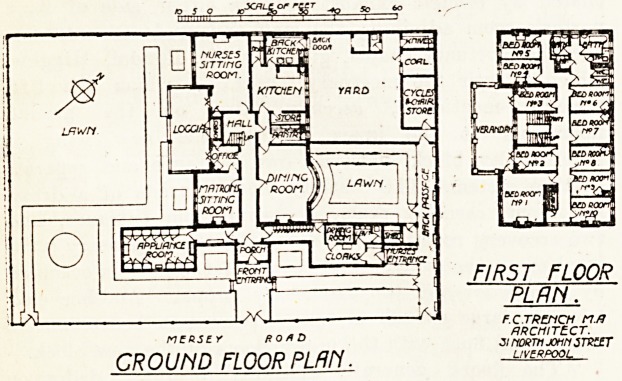# Lady Williamson Memorial Home, Liverpool

**Published:** 1913-08-23

**Authors:** 


### LADY WILLIAMSON MEMORIAL HOME, LIVERPOOL.

THIS institution is a Home or Hostel for Nurses in connection with the Liverpool Queen Victoria District Nursing Association, and was presented to the Association by Sir Archibald Williamson, Bart., M.P., in memory of his late wife.

GROUND FLOOR PLAN. FIRST FLOOR PLAN.

The building is a picturesque and homelike edifice, and provides accommodation for a matron, ten nurses, a visitor, and two servants. The Home faces south-west and south-east, and is provided with a small garden.

On the ground floor, on one side of the front entrance, is a room fitted with store cupboards for containing medicines and appliances, with a sink for hot and cold water, and a glass-topped table. Provision is made here also for sterilising instruments. On the other side of the entrance is a large cloak room fitted with lockers for the nurses, also a drying room and lavatory.

In the main building, facing south-west, will be found the matron's sitting room and her office adjoining, nurses' sitting room, and a spacious open loggia looking on to the garden, which forms a most attractive part of the building. On the other side of the corridor is a dining room, with pantry adjoining, and the kitchen offices. In the yard, approached from the nurses' private entrance, is a large store for bicycles and a store for invalid chairs, also a coal store.

On the first floor are eight nurses's bedrooms, the matron's bedroom, and a bedroom for the use of visitors. Two of these rooms open on to a verandah over the loggia on the ground floor. Bathrooms, sanitary offices, and linen store are also provided on this floor.

On the second floor are four bedrooms, two box rooms, bathroom, and two store cupboards.

The building is well arranged, and is of remarkably attractive appearance, and should prove a most valuable help to the important work of district nursing in Liverpool. The architect is Mr. F. C. Trench, M.A., of Liverpool.

## Figures and Tables

**Figure f1:**